# Crystal structure of bis­[1,3-bis­(2,6-diiso­propyl­phen­yl)imidazol-2-yl­idene]silver(I) chloride tetrahydro­furan monosolvate

**DOI:** 10.1107/S2056989015007525

**Published:** 2015-04-22

**Authors:** Inge Sänger, Hans-Wolfram Lerner, Michael Bolte

**Affiliations:** a Institut für Anorganische Chemie, Goethe-Universität Frankfurt, Max-von-Laue-Strasse 7, 60438 Frankfurt/Main, Germany

**Keywords:** crystal structure, P_4_, NHC, imidazole, 1,3-bis­(2,6-di­methyl­phen­yl)imidazol-2-yl­idene, C—H⋯Cl hydrogen bonds, C—H⋯O hydrogen bonds.

## Abstract

In the title salt, [Ag(C_27_H_36_N_2_)_2_]Cl·C_4_H_8_O, the Ag^I^ atom is coordinated by two 1,3-bis­(2,6-di­methyl­phen­yl)imidazol-2-yl­idene ligands. The imidazole rings are inclined to one another by 46.69 (13)° and the benzene rings in each ligand are almost normal to the imdazole ring to which they are attached, with dihedral angles varying from 82.39 (13) to 88.27 (12)°. There are C—H⋯π inter­actions present in the cation, involving the two ligands, and the solvent mol­ecule is linked to the cation *via* a C—H⋯O hydrogen bond. In the crystal, mol­ecules are linked by trifurcated C—H⋯(Cl,Cl,Cl) hydrogen bonds, forming slabs parallel to (101). One isopropyl group is disordered over two sets of sites with an occupancy ratio of 0.447 (17):0.553 (17) and the THF mol­ecule is disordered over two positions with an occupancy ratio of 0.589 (6):0.411 (6).

## Abstract 2

In the title compound, the silver atom is coordinated by two 1,3-bis­(2,6-di­methyl­phen­yl)imidazol-2-yl­idene ligands, with the imidazole rings inclined to one another by 46.69 (13)°. In the crystal, mol­ecules are linked by trifurcated C—H⋯(Cl,Cl,Cl) hydrogen bonds, forming two-dimensional networks parallel to (010).

## Chemical context   

In the past few decades the reactivity of white phospho­rus towards nucleophilic agents has been studied extensively (Scheer *et al.*, 2010 ▸). Previously, we have reported that the products of the reaction between P_4_ and the tri-*tert*-butyl­silanides (supersilanides) *M*[Si(*t*Bu)_3_] (*M* = Li, Na, K) (Lerner, 2005 ▸) depend strongly on the stoichiometry and solvent (Lorbach *et al.*, 2009 ▸, 2011 ▸). The sodium penta­phosphide Na_2_[P_5_(Si*t*Bu_3_)_3_] was directly accessible by treating P_4_ with four equivalents of the sodium silanide Na(thf)_2_[Si*t*Bu_3_] in benzene (Lerner *et al.*, 2005 ▸). 
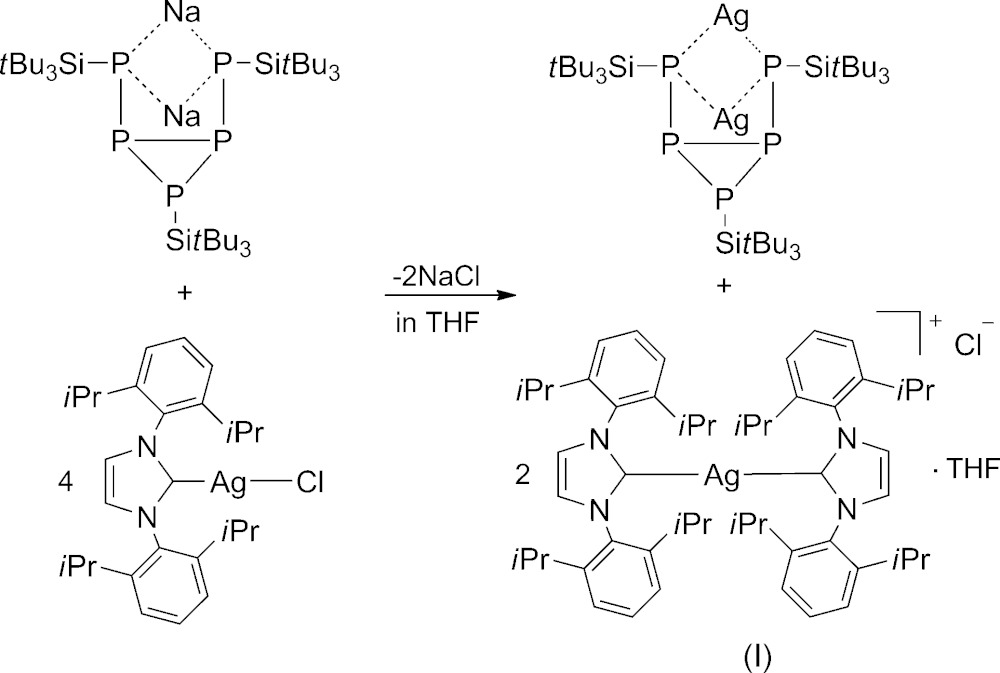


 Recently, we have shown that the penta­phosphenide Na_2_[P_5_(Si*t*Bu_3_)_3_] can be converted into Ag_2_[P_5_(Si*t*Bu_3_)_3_] by a metathesis reaction between Na_2_[P_5_(Si*t*Bu_3_)_3_] and AgOCN (Lerner *et al.*, 2005 ▸). In this paper we present the reaction of Na_2_[P_5_(Si*t*Bu_3_)_3_] with 1,3-bis­(2,6-diiso­propyl­phen­yl)imidazol-2-yliden)silver(I) chloride ([Ag(NHC)Cl]) in a molar ratio of 1:4 in THF which gives Ag_2_[P_5_(Si*t*Bu_3_)_3_] and [Ag(NHC)_2_]Cl. Herein, the crystal structure of one of the two products of this reaction, [Ag(NHC)_2_]Cl·THF, (I), is described.

## Structural commentary   

**Figure 1:**
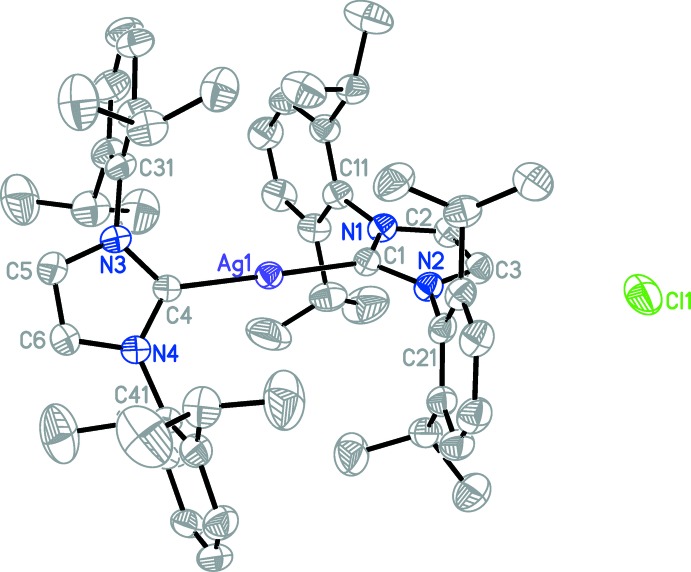
The mol­ecular structure of the title compound (I), with atom labelling. Displacement ellipsoids are drawn at the 50% probability level. Hydrogen atoms and the minor occupied sites of the disordered isopropyl group and the disordered THF mol­ecule have been omitted for clarity.

The title compound (Fig. 1 ▸) crystallizes with discrete bis­(1,3-bis­(2,6-di-iso­propyl­phen­yl)-2,3-di­hydro-1*H*-imidazol-2-ylidene)silver(I) cations, chloride anions and a THF solvent mol­ecule in a 1:1:1 ratio. The Ag atom is bonded to two C atoms with bond lengths Ag1—C4 = 2.103 (2) and Ag1—C1 = 2.1058 (19) Å. The C1—Ag1—C4 bond angle is almost perfectly linear at 179.36 (7)°. The dihedral angle between the two heterocycles is 46.70 (11)°. The two 2,6-di-iso­propyl­phenyl rings (C11–C16 and C21–C26) are inclined to the imdazole ring (N1/N2/C1–C3) by 86.64 (12) and 88.27 (12)°, respectively. In the second ligand, the two 2,6-di-iso­propyl­phenl rings (C31–C36 and C41–C46) are inclined to the imidazole ring (N3/N4/C4–C6) by 82.39 (13) and 83.41 (13)°, respectively. There are also C—H⋯π inter­actions present involving the two ligands (Table 1 ▸).

## Supra­molecular features   

**Figure 2:**
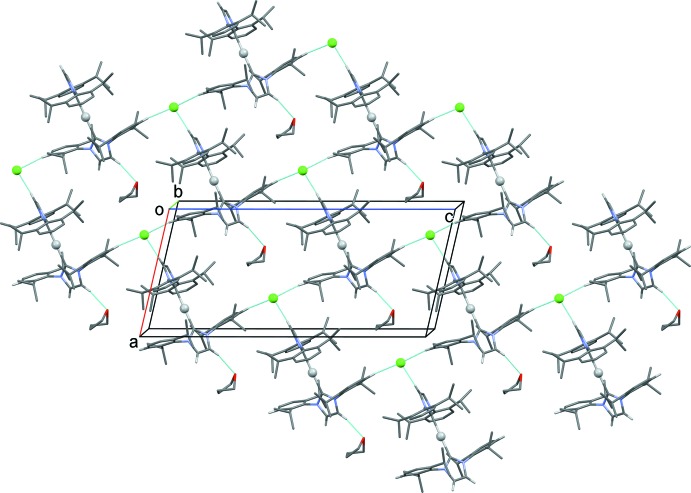
The crystal packing of the title compound (I), viewed along the *b* axis. The C—H⋯Cl and C—H⋯O hydrogen bonds are shown as dashed lines (see Table 1 ▸ for details). Disordered atoms and H atoms not involved in hydrogen bonding have been omitted for clarity (Ag silver ball, Cl green ball).

In the crystal, mol­ecules are bridged by the Cl anions which form C—H⋯Cl⋯H—C hydrogen bonds, forming slabs lying parallel to (101); Table 1 ▸ and Fig. 2 ▸.

## Database survey   

The structures of the same cation but with different anions have been reported, *viz.* bis­[1,3-bis­(2,6-di-iso­propyl­phen­yl)-2,3-di­hydro-1*H*-imidazol-2-yl­idene]silver(I) tetra­chlorido­gallate(III) (I*a*) (Tang *et al.*, 2012 ▸) and bis­[1,3-bis­(2,6-diiso­propyl­phen­yl)imidazol-2-yl­idene]silver hexa­fluorido­anti­mon­ate(V) (I*b*) (Partyka & Deligonul, 2009 ▸). These two structures have a bond angle of exactly 180° at the Ag atom due to symmetry whereas the C—Ag—C angle in the title compound deviates insignificantly from linearity [179.36 (7)°]. The Ag—C distances are also comparable with the values in the title compound [2.103 Å in (I*a*) and 2.128 and 2.129 Å in (I*b*)]. However, while the dihedral angle between the two heterocycles is 46.70 (11)° in the title compound, it is significantly smaller in (I*a*) (32.4°) and (I*b*) (37.8°).

A database search (CSD, Version 5.36, November 2014; Groom & Allen, 2014 ▸) for [1,3-bis­(2,6-diiso­propyl­phen­yl)imidazol-2-yl­idene]silver yielded eight hits with ten fragments. The mean Ag—C bond length in these structures is 2.09 (3) Å. These values agree well with those for the title compound, *viz.* Ag1—C1 = 2.1058 (19) and Ag1—C4 = 2.103 (2) Å.

## Synthesis and crystallization   

A solution of Na_2_[P_5_(Si*t*Bu_3_)_3_] (0.1 mmol) in 1 mL THF was treated with a solution of [Ag(NHC)Cl] (0.21 g, 0.4 mmol) 2 mL THF. The reaction mixture was stirred for 18 h at room temperature. After overlaying the THF solution with cyclo­hexane (6 mL), colourless block-like crystals of the title compound were obtained after 10 days at room temperature (yield: 41%).

## Refinement details   

Crystal data, data collection and structure refinement details are summarized in Table 2 ▸. The C-bound H atoms were fixed geometrically and refined using a riding model approximation: C—H = 0.95–1.00 Å with *U*_iso_(H) = 1.5*U*_eq_(C) for methyl H atoms and 1.2*U*_eq_(C) for other H atoms. One isopropyl group (atoms C481/C482 and C483/C484) is disordered over two sets of sites with an occupancy ratio of 0.447 (17):0.553 (17) while the THF mol­ecule is disordered over two positions with an occupancy ratio of 0.589 (6):0.411 (6). Symmetry-equivalent bond lengths and angles in the two THF sites were restrained to be equal, distance C73′⋯C75′ was restrained to 2.30 (1) Å, and the displacement parameters of the C atoms were restrained to an isotropic behaviour.

## Supplementary Material

Crystal structure: contains datablock(s) I, Global. DOI: 10.1107/S2056989015007525/su5115sup1.cif

Structure factors: contains datablock(s) I. DOI: 10.1107/S2056989015007525/su5115Isup2.hkl

CCDC reference: 1060013

Additional supporting information:  crystallographic information; 3D view; checkCIF report

## Figures and Tables

**Table 1:** Hydrogen-bond geometry (, ) *Cg*1, *Cg*2 and *Cg*3 are the centroids of rings C31C26, C11C16 and C21C26, respectively.

*D*H*A*	*D*H	H*A*	*D* *A*	*D*H*A*
C5H5O71	0.95	2.42	3.292(5)	153
C3H3Cl1	0.95	2.51	3.422(2)	161
C35H35Cl1^i^	0.95	2.68	3.627(3)	174
C43H43Cl1^ii^	0.95	2.64	3.562(2)	163
C171H17*B* *Cg*1	0.98	2.81	3.532(4)	131
C372H37*C* *Cg*2	0.98	2.94	3.613(4)	126
C481H48*A* *Cg*3	0.98	2.98	3.840(12)	147

Symmetry codes: (i) 
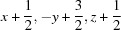
; (ii) 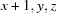.

**Table 2:** Experimental details

Crystal data	
Chemical formula	[Ag(C_27_H_36_N_2_)_2_]ClC_4_H_8_O
*M*_r_	992.58
Crystal system, space group	Monoclinic, *P*2_1_/*n*
Temperature (K)	173
*a*, *b*, *c* ()	11.9302(3), 18.3390(5), 26.0144(6)
()	103.068(2)
*V* (^3^)	5544.2(2)
*Z*	4
Radiation type	Mo *K*
(mm^1^)	0.45
Crystal size (mm)	0.31 0.27 0.26

Data collection	
Diffractometer	Stoe IPDS 2
Absorption correction	Multi-scan (*X-AREA*; Stoe Cie, 2001 ▸)
*T*_min_, *T*_max_	0.571, 1.000
No. of measured, independent and observed [*I* > 2(*I*)] reflections	111561, 15949, 13800
*R*_int_	0.075
(sin /)_max_ (^1^)	0.705

Refinement	
*R*[*F* ^2^ > 2(*F* ^2^)], *wR*(*F* ^2^), *S*	0.048, 0.121, 1.05
No. of reflections	15949
No. of parameters	651
No. of restraints	75
H-atom treatment	H-atom parameters constrained
_max_, _min_ (e ^3^)	1.03, 1.37

Computer programs: *X-AREA* and *X-RED32* (Stoe Cie, 2001 ▸), *SHELXS97* and *XP* (Sheldrick, 2008 ▸), *SHELXL2014* (Sheldrick, 2015 ▸), *PLATON* (Spek, 2009 ▸) and *publCIF* (Westrip, 2010 ▸).

## supplementary crystallographic information

### Crystal data

**Table:**

[Ag(C_27_H_36_N_2_)_2_]Cl·C_4_H_8_O	*F*(000) = 2112
*M**_r_* = 992.58	*D*_x_ = 1.189 Mg m^−^^3^
Monoclinic, *P*2_1_/*n*	Mo *K*α radiation, λ = 0.71073 Å
*a* = 11.9302 (3) Å	Cell parameters from 115747 reflections
*b* = 18.3390 (5) Å	θ = 2.0–30.3°
*c* = 26.0144 (6) Å	µ = 0.45 mm^−^^1^
β = 103.068 (2)°	*T* = 173 K
*V* = 5544.2 (2) Å^3^	Block, colourless
*Z* = 4	0.31 × 0.27 × 0.26 mm

### Data collection

**Table:**

Stoe IPDS 2 diffractometer	15949 independent reflections
Radiation source: fine-focus sealed tube	13800 reflections with *I* > 2σ(*I*)
Plane graphite monochromator	*R*_int_ = 0.075
ω scans	θ_max_ = 30.1°, θ_min_ = 2.1°
Absorption correction: multi-scan (*X-AREA*; Stoe & Cie, 2001)	*h* = −16→16
*T*_min_ = 0.571, *T*_max_ = 1.000	*k* = −25→25
111561 measured reflections	*l* = −36→36

### Refinement

**Table:**

Refinement on *F*^2^	Primary atom site location: structure-invariant direct methods
Least-squares matrix: full	Hydrogen site location: inferred from neighbouring sites
*R*[*F*^2^ > 2σ(*F*^2^)] = 0.048	H-atom parameters constrained
*wR*(*F*^2^) = 0.121	*w* = 1/[σ^2^(*F*_o_^2^) + (0.0596*P*)^2^ + 3.8737*P*] where *P* = (*F*_o_^2^ + 2*F*_c_^2^)/3
*S* = 1.05	(Δ/σ)_max_ = 0.001
15949 reflections	Δρ_max_ = 1.03 e Å^−^^3^
651 parameters	Δρ_min_ = −1.37 e Å^−^^3^
75 restraints	

### Special details

**Table:**

Geometry. All e.s.d.'s (except the e.s.d. in the dihedral angle between two l.s. planes) are estimated using the full covariance matrix. The cell e.s.d.'s are taken into account individually in the estimation of e.s.d.'s in distances, angles and torsion angles; correlations between e.s.d.'s in cell parameters are only used when they are defined by crystal symmetry. An approximate (isotropic) treatment of cell e.s.d.'s is used for estimating e.s.d.'s involving l.s. planes.

### Fractional atomic coordinates and isotropic or equivalent isotropic displacement parameters (Å^2^)

**Table:**

	*x*	*y*	*z*	*U*_iso_*/*U*_eq_	Occ. (<1)
Cl1	−0.25283 (7)	0.79025 (8)	0.41564 (3)	0.0832 (3)	
Ag1	0.32700 (2)	0.73977 (2)	0.61445 (2)	0.02859 (5)	
N1	0.10444 (15)	0.66141 (9)	0.55242 (7)	0.0324 (3)	
N2	0.09066 (15)	0.77562 (9)	0.53662 (7)	0.0318 (3)	
C1	0.16299 (17)	0.72485 (11)	0.56468 (7)	0.0295 (4)	
C2	−0.00005 (19)	0.67258 (12)	0.51718 (8)	0.0366 (4)	
H2	−0.0549	0.6362	0.5028	0.044*	
C3	−0.00911 (19)	0.74443 (12)	0.50725 (9)	0.0365 (4)	
H3	−0.0714	0.7689	0.4846	0.044*	
C11	0.14323 (19)	0.58950 (11)	0.57077 (8)	0.0339 (4)	
C12	0.11632 (19)	0.56315 (12)	0.61711 (9)	0.0376 (4)	
C13	0.1487 (2)	0.49169 (14)	0.63150 (11)	0.0472 (5)	
H13	0.1342	0.4726	0.6633	0.057*	
C14	0.2013 (3)	0.44784 (14)	0.60066 (12)	0.0512 (6)	
H14	0.2208	0.3989	0.6109	0.061*	
C15	0.2255 (2)	0.47539 (13)	0.55510 (11)	0.0472 (5)	
H15	0.2613	0.4449	0.5340	0.057*	
C16	0.1985 (2)	0.54696 (12)	0.53932 (9)	0.0397 (5)	
C17	0.0557 (2)	0.61055 (14)	0.65057 (9)	0.0418 (5)	
H17	0.0084	0.6472	0.6266	0.050*	
C18	0.2273 (3)	0.57670 (15)	0.48954 (10)	0.0484 (6)	
H18	0.2044	0.6292	0.4862	0.058*	
C21	0.11102 (17)	0.85337 (11)	0.53718 (8)	0.0315 (4)	
C22	0.16141 (18)	0.88315 (12)	0.49827 (8)	0.0355 (4)	
C23	0.1789 (2)	0.95834 (13)	0.49946 (10)	0.0414 (5)	
H23	0.2142	0.9802	0.4740	0.050*	
C24	0.1459 (2)	1.00192 (12)	0.53687 (10)	0.0421 (5)	
H24	0.1595	1.0530	0.5372	0.051*	
C25	0.0930 (2)	0.97107 (12)	0.57377 (9)	0.0389 (4)	
H25	0.0694	1.0015	0.5989	0.047*	
C26	0.07386 (18)	0.89620 (11)	0.57481 (8)	0.0342 (4)	
C27	0.1978 (2)	0.83601 (14)	0.45695 (9)	0.0421 (5)	
H27	0.1554	0.7887	0.4552	0.050*	
C28	0.0168 (2)	0.86275 (13)	0.61588 (9)	0.0408 (5)	
H28	−0.0058	0.8116	0.6047	0.049*	
N3	0.53162 (16)	0.72843 (10)	0.71425 (7)	0.0346 (4)	
N4	0.57794 (15)	0.79569 (11)	0.65566 (7)	0.0350 (4)	
C4	0.48997 (17)	0.75476 (11)	0.66489 (8)	0.0292 (3)	
C5	0.6422 (2)	0.75307 (16)	0.73539 (10)	0.0474 (6)	
H5	0.6884	0.7420	0.7693	0.057*	
C6	0.6713 (2)	0.79563 (16)	0.69851 (10)	0.0476 (6)	
H6	0.7420	0.8208	0.7012	0.057*	
C31	0.47404 (19)	0.67686 (12)	0.74141 (8)	0.0346 (4)	
C32	0.4827 (2)	0.60301 (13)	0.72966 (9)	0.0421 (5)	
C33	0.4296 (3)	0.55367 (14)	0.75715 (11)	0.0501 (6)	
H33	0.4320	0.5030	0.7498	0.060*	
C34	0.3733 (2)	0.57756 (15)	0.79501 (10)	0.0492 (6)	
H34	0.3372	0.5431	0.8133	0.059*	
C35	0.3688 (2)	0.65110 (14)	0.80671 (9)	0.0444 (5)	
H35	0.3312	0.6664	0.8334	0.053*	
C36	0.4192 (2)	0.70272 (12)	0.77967 (8)	0.0377 (4)	
C37	0.5505 (3)	0.57671 (16)	0.69008 (12)	0.0538 (6)	
H37	0.5788	0.6208	0.6743	0.065*	
C38	0.4136 (2)	0.78324 (14)	0.79234 (10)	0.0451 (5)	
H38	0.4406	0.8116	0.7646	0.054*	
C41	0.58009 (17)	0.83616 (12)	0.60799 (8)	0.0333 (4)	
C42	0.61299 (18)	0.79945 (13)	0.56671 (9)	0.0370 (4)	
C43	0.6249 (2)	0.84112 (15)	0.52329 (9)	0.0428 (5)	
H43	0.6468	0.8182	0.4943	0.051*	
C44	0.6050 (2)	0.91521 (15)	0.52225 (10)	0.0477 (6)	
H44	0.6150	0.9430	0.4928	0.057*	
C45	0.5707 (2)	0.94981 (14)	0.56344 (10)	0.0465 (5)	
H45	0.5560	1.0008	0.5615	0.056*	
C46	0.55756 (19)	0.91082 (13)	0.60776 (9)	0.0389 (4)	
C47	0.6370 (2)	0.71821 (15)	0.56861 (11)	0.0470 (5)	
H47	0.5884	0.6948	0.5906	0.056*	
C48	0.5198 (2)	0.94851 (15)	0.65304 (11)	0.0484 (5)	
H48	0.5085	0.9087	0.6776	0.058*	0.447 (17)
H48'	0.5559	0.9219	0.6862	0.058*	0.553 (17)
C171	0.1420 (3)	0.65204 (18)	0.69207 (12)	0.0595 (7)	
H17A	0.1009	0.6820	0.7130	0.089*	
H17B	0.1920	0.6174	0.7152	0.089*	
H17C	0.1888	0.6835	0.6748	0.089*	
C172	−0.0252 (3)	0.5682 (2)	0.67752 (13)	0.0685 (9)	
H17D	−0.0812	0.5412	0.6509	0.103*	
H17E	0.0196	0.5338	0.7030	0.103*	
H17F	−0.0658	0.6022	0.6959	0.103*	
C181	0.1623 (4)	0.5374 (3)	0.44116 (13)	0.0885 (13)	
H18A	0.0795	0.5405	0.4395	0.133*	
H18B	0.1791	0.5600	0.4096	0.133*	
H18C	0.1858	0.4861	0.4429	0.133*	
C271	0.1680 (3)	0.87042 (18)	0.40178 (11)	0.0579 (7)	
H27A	0.1931	0.8380	0.3766	0.087*	
H27B	0.0846	0.8778	0.3908	0.087*	
H27C	0.2072	0.9175	0.4026	0.087*	
C272	0.3262 (3)	0.8194 (2)	0.47309 (13)	0.0701 (9)	
H27D	0.3483	0.7890	0.4461	0.105*	
H27E	0.3697	0.8652	0.4767	0.105*	
H27F	0.3430	0.7935	0.5069	0.105*	
C281	0.1018 (3)	0.85940 (17)	0.66940 (10)	0.0547 (7)	
H28A	0.0640	0.8378	0.6955	0.082*	
H28B	0.1681	0.8295	0.6665	0.082*	
H28C	0.1278	0.9088	0.6806	0.082*	
C381	0.4926 (3)	0.80103 (19)	0.84580 (14)	0.0658 (8)	
H38A	0.4875	0.8532	0.8531	0.099*	
H38B	0.4687	0.7727	0.8735	0.099*	
H38C	0.5721	0.7886	0.8451	0.099*	
C382	0.2906 (2)	0.80671 (16)	0.79172 (11)	0.0521 (6)	
H38D	0.2895	0.8588	0.8000	0.078*	
H38E	0.2415	0.7977	0.7567	0.078*	
H38F	0.2618	0.7787	0.8181	0.078*	
C471	0.7617 (3)	0.7022 (2)	0.5937 (2)	0.0959 (15)	
H47A	0.7746	0.6494	0.5944	0.144*	
H47B	0.8112	0.7257	0.5732	0.144*	
H47C	0.7803	0.7212	0.6299	0.144*	
C472	0.6065 (4)	0.6833 (2)	0.51324 (15)	0.0744 (10)	
H47D	0.5257	0.6935	0.4967	0.112*	
H47E	0.6559	0.7039	0.4915	0.112*	
H47F	0.6184	0.6305	0.5164	0.112*	
C282	−0.0920 (3)	0.90368 (19)	0.62077 (14)	0.0614 (7)	
H28D	−0.1255	0.8801	0.6476	0.092*	
H28E	−0.0723	0.9543	0.6311	0.092*	
H28F	−0.1478	0.9028	0.5867	0.092*	
C372	0.4758 (3)	0.5337 (2)	0.64546 (13)	0.0747 (10)	
H37A	0.5222	0.5178	0.6209	0.112*	
H37B	0.4443	0.4909	0.6599	0.112*	
H37C	0.4126	0.5645	0.6267	0.112*	
C371	0.6557 (3)	0.5331 (2)	0.71798 (18)	0.0836 (12)	
H37D	0.7019	0.5625	0.7465	0.125*	
H37E	0.6306	0.4884	0.7327	0.125*	
H37F	0.7021	0.5204	0.6926	0.125*	
C182	0.3550 (4)	0.5721 (4)	0.49240 (18)	0.130 (3)	
H18D	0.3970	0.5978	0.5240	0.195*	
H18E	0.3788	0.5209	0.4942	0.195*	
H18F	0.3721	0.5948	0.4609	0.195*	
C481	0.4036 (10)	0.9851 (11)	0.6354 (4)	0.082 (4)	0.447 (17)
H48A	0.3478	0.9501	0.6159	0.123*	0.447 (17)
H48B	0.3774	1.0023	0.6665	0.123*	0.447 (17)
H48C	0.4102	1.0267	0.6126	0.123*	0.447 (17)
C482	0.6139 (10)	0.9942 (9)	0.6833 (6)	0.086 (5)	0.447 (17)
H48D	0.5884	1.0182	0.7123	0.130*	0.447 (17)
H48E	0.6810	0.9636	0.6976	0.130*	0.447 (17)
H48F	0.6348	1.0313	0.6600	0.130*	0.447 (17)
C483	0.3931 (7)	0.9449 (6)	0.6467 (4)	0.070 (2)	0.553 (17)
H48G	0.3716	0.9696	0.6765	0.104*	0.553 (17)
H48H	0.3553	0.9689	0.6137	0.104*	0.553 (17)
H48I	0.3689	0.8937	0.6458	0.104*	0.553 (17)
C484	0.5609 (11)	1.0302 (5)	0.6600 (4)	0.077 (3)	0.553 (17)
H48J	0.6446	1.0323	0.6642	0.116*	0.553 (17)
H48K	0.5234	1.0583	0.6288	0.116*	0.553 (17)
H48L	0.5403	1.0508	0.6914	0.116*	0.553 (17)
O71	0.8401 (3)	0.7674 (2)	0.84592 (17)	0.0709 (14)	0.589 (6)
C72	0.9183 (7)	0.7094 (4)	0.8373 (3)	0.081 (2)	0.589 (6)
H72A	0.9685	0.6941	0.8712	0.097*	0.589 (6)
H72B	0.8739	0.6665	0.8208	0.097*	0.589 (6)
C73	0.9877 (7)	0.7388 (4)	0.8023 (3)	0.0728 (18)	0.589 (6)
H73A	0.9638	0.7172	0.7666	0.087*	0.589 (6)
H73B	1.0703	0.7281	0.8164	0.087*	0.589 (6)
C74	0.9636 (9)	0.8317 (5)	0.7999 (4)	0.099 (3)	0.589 (6)
H74A	1.0309	0.8617	0.7969	0.119*	0.589 (6)
H74B	0.8927	0.8473	0.7745	0.119*	0.589 (6)
C75	0.9500 (11)	0.8200 (7)	0.8552 (5)	0.150 (5)	0.589 (6)
H75A	0.9353	0.8665	0.8718	0.180*	0.589 (6)
H75B	1.0187	0.7962	0.8773	0.180*	0.589 (6)
O71'	0.9421 (7)	0.7754 (4)	0.7663 (2)	0.084 (2)	0.411 (6)
C72'	0.9671 (10)	0.7119 (6)	0.8015 (4)	0.077 (3)	0.411 (6)
H72C	1.0503	0.7004	0.8087	0.092*	0.411 (6)
H72D	0.9240	0.6689	0.7844	0.092*	0.411 (6)
C73'	0.9325 (10)	0.7283 (5)	0.8529 (4)	0.075 (3)	0.411 (6)
H73C	0.9966	0.7197	0.8838	0.090*	0.411 (6)
H73D	0.8645	0.6996	0.8565	0.090*	0.411 (6)
C74'	0.8976 (9)	0.8296 (5)	0.8429 (4)	0.072 (2)	0.411 (6)
H74C	0.8248	0.8412	0.8174	0.087*	0.411 (6)
H74D	0.9084	0.8590	0.8756	0.087*	0.411 (6)
C75'	1.0046 (8)	0.8220 (5)	0.8193 (4)	0.075 (3)	0.411 (6)
H75C	1.0672	0.7942	0.8426	0.089*	0.411 (6)
H75D	1.0340	0.8698	0.8105	0.089*	0.411 (6)

### Atomic displacement parameters (Å^2^)

**Table:**

	*U*^11^	*U*^22^	*U*^33^	*U*^12^	*U*^13^	*U*^23^
Cl1	0.0488 (4)	0.1689 (10)	0.0337 (3)	0.0122 (5)	0.0133 (3)	0.0032 (4)
Ag1	0.03022 (8)	0.02622 (8)	0.02919 (8)	−0.00159 (5)	0.00643 (5)	0.00055 (5)
N1	0.0379 (9)	0.0265 (8)	0.0320 (8)	−0.0028 (6)	0.0063 (6)	−0.0009 (6)
N2	0.0345 (8)	0.0266 (8)	0.0328 (8)	−0.0026 (6)	0.0046 (6)	0.0013 (6)
C1	0.0306 (9)	0.0266 (9)	0.0300 (8)	−0.0016 (7)	0.0041 (7)	−0.0001 (6)
C2	0.0363 (10)	0.0338 (10)	0.0372 (10)	−0.0064 (8)	0.0028 (8)	−0.0032 (8)
C3	0.0333 (10)	0.0365 (11)	0.0366 (10)	−0.0042 (8)	0.0016 (8)	−0.0008 (8)
C11	0.0384 (10)	0.0244 (9)	0.0386 (10)	−0.0052 (7)	0.0081 (8)	−0.0022 (7)
C12	0.0384 (10)	0.0343 (10)	0.0405 (10)	−0.0044 (8)	0.0100 (8)	0.0016 (8)
C13	0.0560 (14)	0.0375 (12)	0.0501 (13)	−0.0036 (10)	0.0163 (11)	0.0092 (10)
C14	0.0618 (16)	0.0290 (11)	0.0637 (16)	0.0017 (10)	0.0164 (13)	0.0068 (10)
C15	0.0563 (14)	0.0310 (11)	0.0577 (14)	0.0002 (10)	0.0197 (11)	−0.0060 (10)
C16	0.0479 (12)	0.0307 (10)	0.0426 (11)	−0.0047 (9)	0.0144 (9)	−0.0052 (8)
C17	0.0444 (12)	0.0458 (12)	0.0371 (10)	0.0009 (9)	0.0132 (9)	0.0009 (9)
C18	0.0662 (16)	0.0420 (13)	0.0424 (12)	−0.0014 (11)	0.0235 (11)	−0.0041 (9)
C21	0.0321 (9)	0.0260 (9)	0.0334 (9)	−0.0017 (7)	0.0008 (7)	0.0028 (7)
C22	0.0343 (10)	0.0331 (10)	0.0381 (10)	−0.0016 (8)	0.0060 (8)	0.0034 (8)
C23	0.0421 (11)	0.0356 (11)	0.0458 (11)	−0.0059 (9)	0.0081 (9)	0.0085 (9)
C24	0.0455 (12)	0.0280 (10)	0.0489 (12)	−0.0042 (8)	0.0021 (9)	0.0050 (8)
C25	0.0441 (11)	0.0307 (10)	0.0390 (10)	−0.0008 (8)	0.0032 (9)	−0.0013 (8)
C26	0.0372 (10)	0.0304 (10)	0.0327 (9)	−0.0011 (8)	0.0031 (7)	0.0014 (7)
C27	0.0436 (12)	0.0428 (12)	0.0419 (11)	−0.0011 (9)	0.0139 (9)	0.0036 (9)
C28	0.0519 (13)	0.0335 (11)	0.0389 (10)	−0.0019 (9)	0.0138 (9)	−0.0003 (8)
N3	0.0350 (9)	0.0377 (9)	0.0317 (8)	−0.0020 (7)	0.0089 (7)	0.0065 (7)
N4	0.0310 (8)	0.0413 (10)	0.0330 (8)	−0.0016 (7)	0.0078 (6)	0.0075 (7)
C4	0.0287 (9)	0.0314 (9)	0.0281 (8)	−0.0006 (7)	0.0079 (7)	0.0020 (6)
C5	0.0362 (11)	0.0635 (16)	0.0386 (11)	−0.0074 (10)	0.0001 (9)	0.0128 (10)
C6	0.0309 (10)	0.0644 (16)	0.0444 (12)	−0.0091 (10)	0.0019 (9)	0.0142 (11)
C31	0.0385 (10)	0.0357 (10)	0.0294 (9)	−0.0011 (8)	0.0077 (7)	0.0077 (7)
C32	0.0485 (12)	0.0365 (11)	0.0424 (11)	0.0036 (9)	0.0123 (9)	0.0036 (9)
C33	0.0643 (16)	0.0338 (12)	0.0525 (14)	−0.0004 (11)	0.0137 (12)	0.0091 (10)
C34	0.0592 (15)	0.0440 (13)	0.0452 (12)	−0.0054 (11)	0.0138 (11)	0.0157 (10)
C35	0.0532 (13)	0.0489 (13)	0.0339 (10)	−0.0022 (10)	0.0154 (9)	0.0079 (9)
C36	0.0458 (11)	0.0383 (11)	0.0296 (9)	−0.0004 (9)	0.0096 (8)	0.0042 (8)
C37	0.0631 (16)	0.0443 (14)	0.0586 (15)	0.0085 (12)	0.0236 (13)	−0.0011 (11)
C38	0.0557 (14)	0.0392 (12)	0.0428 (11)	−0.0013 (10)	0.0160 (10)	−0.0016 (9)
C41	0.0292 (9)	0.0373 (10)	0.0342 (9)	−0.0045 (7)	0.0088 (7)	0.0069 (7)
C42	0.0312 (9)	0.0414 (11)	0.0407 (10)	−0.0050 (8)	0.0131 (8)	0.0021 (8)
C43	0.0371 (11)	0.0554 (14)	0.0385 (11)	−0.0078 (10)	0.0138 (9)	0.0034 (9)
C44	0.0463 (13)	0.0549 (15)	0.0415 (11)	−0.0114 (11)	0.0088 (10)	0.0160 (10)
C45	0.0477 (13)	0.0385 (12)	0.0495 (13)	−0.0048 (10)	0.0029 (10)	0.0106 (10)
C46	0.0367 (10)	0.0390 (11)	0.0396 (10)	−0.0034 (8)	0.0054 (8)	0.0016 (8)
C47	0.0448 (12)	0.0410 (12)	0.0613 (15)	0.0012 (10)	0.0246 (11)	0.0009 (11)
C48	0.0494 (13)	0.0467 (13)	0.0474 (13)	0.0018 (11)	0.0075 (10)	−0.0053 (10)
C171	0.0633 (17)	0.0628 (18)	0.0582 (16)	−0.0173 (14)	0.0260 (13)	−0.0195 (13)
C172	0.070 (2)	0.081 (2)	0.0649 (18)	−0.0287 (17)	0.0377 (16)	−0.0225 (16)
C181	0.112 (3)	0.105 (3)	0.0439 (16)	−0.030 (3)	0.0069 (18)	−0.0029 (17)
C271	0.0677 (18)	0.0616 (17)	0.0425 (13)	0.0044 (14)	0.0083 (12)	0.0018 (12)
C272	0.0559 (17)	0.104 (3)	0.0530 (16)	0.0265 (17)	0.0175 (13)	0.0066 (16)
C281	0.0689 (18)	0.0589 (16)	0.0372 (12)	0.0109 (13)	0.0135 (11)	0.0050 (11)
C381	0.0578 (17)	0.0581 (18)	0.074 (2)	0.0042 (14)	−0.0018 (15)	−0.0213 (15)
C382	0.0568 (15)	0.0507 (14)	0.0454 (13)	0.0080 (12)	0.0045 (11)	−0.0065 (11)
C471	0.060 (2)	0.067 (2)	0.151 (4)	0.0223 (18)	0.003 (2)	−0.012 (3)
C472	0.094 (3)	0.0565 (19)	0.082 (2)	−0.0096 (17)	0.038 (2)	−0.0203 (16)
C282	0.0504 (15)	0.0671 (19)	0.0712 (18)	0.0040 (13)	0.0228 (13)	0.0133 (15)
C372	0.081 (2)	0.087 (3)	0.0560 (17)	0.018 (2)	0.0150 (16)	−0.0169 (16)
C371	0.060 (2)	0.092 (3)	0.097 (3)	0.0220 (19)	0.0157 (19)	−0.013 (2)
C182	0.071 (3)	0.252 (8)	0.072 (3)	−0.036 (4)	0.028 (2)	0.043 (4)
C481	0.065 (6)	0.108 (11)	0.072 (6)	0.038 (7)	0.013 (4)	−0.012 (6)
C482	0.078 (6)	0.094 (9)	0.089 (8)	−0.017 (6)	0.021 (5)	−0.052 (7)
C483	0.055 (3)	0.062 (5)	0.094 (6)	0.007 (3)	0.024 (3)	−0.023 (4)
C484	0.101 (7)	0.056 (4)	0.077 (5)	−0.022 (4)	0.024 (5)	−0.023 (4)
O71	0.051 (2)	0.092 (3)	0.074 (3)	0.0094 (19)	0.0219 (18)	0.027 (2)
C72	0.081 (2)	0.081 (2)	0.080 (2)	0.0009 (10)	0.0189 (11)	0.0004 (10)
C73	0.072 (2)	0.074 (2)	0.073 (2)	0.0001 (10)	0.0175 (10)	0.0006 (10)
C74	0.099 (3)	0.099 (3)	0.099 (3)	−0.0011 (10)	0.0225 (11)	−0.0008 (10)
C75	0.151 (5)	0.150 (5)	0.150 (5)	−0.0003 (10)	0.0333 (15)	−0.0001 (10)
O71'	0.098 (5)	0.090 (5)	0.060 (4)	0.000 (4)	0.010 (3)	0.005 (3)
C72'	0.077 (3)	0.077 (3)	0.077 (3)	−0.0005 (10)	0.0180 (12)	0.0006 (10)
C73'	0.075 (3)	0.075 (3)	0.074 (3)	0.0009 (10)	0.0174 (12)	−0.0001 (10)
C74'	0.072 (3)	0.073 (3)	0.072 (3)	−0.0003 (10)	0.0173 (11)	−0.0012 (10)
C75'	0.074 (3)	0.074 (3)	0.076 (3)	−0.0002 (10)	0.0178 (11)	−0.0001 (10)

### Geometric parameters (Å, º)

**Table:**

Ag1—C4	2.103 (2)	C48—C483	1.484 (8)
Ag1—C1	2.1058 (19)	C48—C481	1.516 (10)
N1—C1	1.357 (2)	C48—C484	1.574 (7)
N1—C2	1.385 (3)	C48—H48	1.0000
N1—C11	1.443 (3)	C48—H48'	1.0000
N2—C1	1.364 (3)	C171—H17A	0.9800
N2—C3	1.384 (3)	C171—H17B	0.9800
N2—C21	1.446 (3)	C171—H17C	0.9800
C2—C3	1.342 (3)	C172—H17D	0.9800
C2—H2	0.9500	C172—H17E	0.9800
C3—H3	0.9500	C172—H17F	0.9800
C11—C16	1.399 (3)	C181—H18A	0.9800
C11—C12	1.402 (3)	C181—H18B	0.9800
C12—C13	1.393 (3)	C181—H18C	0.9800
C12—C17	1.524 (3)	C271—H27A	0.9800
C13—C14	1.383 (4)	C271—H27B	0.9800
C13—H13	0.9500	C271—H27C	0.9800
C14—C15	1.378 (4)	C272—H27D	0.9800
C14—H14	0.9500	C272—H27E	0.9800
C15—C16	1.391 (3)	C272—H27F	0.9800
C15—H15	0.9500	C281—H28A	0.9800
C16—C18	1.514 (3)	C281—H28B	0.9800
C17—C171	1.517 (4)	C281—H28C	0.9800
C17—C172	1.527 (4)	C381—H38A	0.9800
C17—H17	1.0000	C381—H38B	0.9800
C18—C181	1.504 (4)	C381—H38C	0.9800
C18—C182	1.511 (5)	C382—H38D	0.9800
C18—H18	1.0000	C382—H38E	0.9800
C21—C22	1.400 (3)	C382—H38F	0.9800
C21—C26	1.403 (3)	C471—H47A	0.9800
C22—C23	1.394 (3)	C471—H47B	0.9800
C22—C27	1.517 (3)	C471—H47C	0.9800
C23—C24	1.384 (4)	C472—H47D	0.9800
C23—H23	0.9500	C472—H47E	0.9800
C24—C25	1.383 (3)	C472—H47F	0.9800
C24—H24	0.9500	C282—H28D	0.9800
C25—C26	1.393 (3)	C282—H28E	0.9800
C25—H25	0.9500	C282—H28F	0.9800
C26—C28	1.520 (3)	C372—H37A	0.9800
C27—C272	1.525 (4)	C372—H37B	0.9800
C27—C271	1.534 (4)	C372—H37C	0.9800
C27—H27	1.0000	C371—H37D	0.9800
C28—C281	1.527 (4)	C371—H37E	0.9800
C28—C282	1.528 (4)	C371—H37F	0.9800
C28—H28	1.0000	C182—H18D	0.9800
N3—C4	1.356 (2)	C182—H18E	0.9800
N3—C5	1.386 (3)	C182—H18F	0.9800
N3—C31	1.444 (3)	C481—H48A	0.9800
N4—C4	1.355 (3)	C481—H48B	0.9800
N4—C6	1.387 (3)	C481—H48C	0.9800
N4—C41	1.451 (2)	C482—H48D	0.9800
C5—C6	1.342 (3)	C482—H48E	0.9800
C5—H5	0.9500	C482—H48F	0.9800
C6—H6	0.9500	C483—H48G	0.9800
C31—C36	1.392 (3)	C483—H48H	0.9800
C31—C32	1.397 (3)	C483—H48I	0.9800
C32—C33	1.392 (3)	C484—H48J	0.9800
C32—C37	1.525 (4)	C484—H48K	0.9800
C33—C34	1.383 (4)	C484—H48L	0.9800
C33—H33	0.9500	O71—C72	1.465 (8)
C34—C35	1.386 (4)	O71—C75	1.602 (11)
C34—H34	0.9500	C72—C73	1.464 (9)
C35—C36	1.394 (3)	C72—H72A	0.9900
C35—H35	0.9500	C72—H72B	0.9900
C36—C38	1.518 (3)	C73—C74	1.727 (11)
C37—C372	1.515 (5)	C73—H73A	0.9900
C37—C371	1.526 (5)	C73—H73B	0.9900
C37—H37	1.0000	C74—C75	1.499 (11)
C38—C382	1.526 (4)	C74—H74A	0.9900
C38—C381	1.529 (4)	C74—H74B	0.9900
C38—H38	1.0000	C75—H75A	0.9900
C41—C46	1.395 (3)	C75—H75B	0.9900
C41—C42	1.397 (3)	O71'—C72'	1.472 (11)
C42—C43	1.397 (3)	O71'—C75'	1.651 (10)
C42—C47	1.516 (4)	C72'—C73'	1.515 (11)
C43—C44	1.378 (4)	C72'—H72C	0.9900
C43—H43	0.9500	C72'—H72D	0.9900
C44—C45	1.384 (4)	C73'—C74'	1.908 (12)
C44—H44	0.9500	C73'—H73C	0.9900
C45—C46	1.395 (3)	C73'—H73D	0.9900
C45—H45	0.9500	C74'—C75'	1.543 (11)
C46—C48	1.519 (3)	C74'—H74C	0.9900
C47—C471	1.512 (4)	C74'—H74D	0.9900
C47—C472	1.543 (4)	C75'—H75C	0.9900
C47—H47	1.0000	C75'—H75D	0.9900
C48—C482	1.478 (9)		
			
C4—Ag1—C1	179.36 (7)	C17—C171—H17B	109.5
C1—N1—C2	111.53 (17)	H17A—C171—H17B	109.5
C1—N1—C11	126.59 (17)	C17—C171—H17C	109.5
C2—N1—C11	121.83 (17)	H17A—C171—H17C	109.5
C1—N2—C3	111.98 (17)	H17B—C171—H17C	109.5
C1—N2—C21	125.69 (17)	C17—C172—H17D	109.5
C3—N2—C21	122.31 (17)	C17—C172—H17E	109.5
N1—C1—N2	103.28 (16)	H17D—C172—H17E	109.5
N1—C1—Ag1	127.82 (14)	C17—C172—H17F	109.5
N2—C1—Ag1	128.90 (14)	H17D—C172—H17F	109.5
C3—C2—N1	107.07 (18)	H17E—C172—H17F	109.5
C3—C2—H2	126.5	C18—C181—H18A	109.5
N1—C2—H2	126.5	C18—C181—H18B	109.5
C2—C3—N2	106.12 (19)	H18A—C181—H18B	109.5
C2—C3—H3	126.9	C18—C181—H18C	109.5
N2—C3—H3	126.9	H18A—C181—H18C	109.5
C16—C11—C12	122.7 (2)	H18B—C181—H18C	109.5
C16—C11—N1	118.33 (19)	C27—C271—H27A	109.5
C12—C11—N1	118.76 (19)	C27—C271—H27B	109.5
C13—C12—C11	116.9 (2)	H27A—C271—H27B	109.5
C13—C12—C17	121.4 (2)	C27—C271—H27C	109.5
C11—C12—C17	121.7 (2)	H27A—C271—H27C	109.5
C14—C13—C12	121.7 (2)	H27B—C271—H27C	109.5
C14—C13—H13	119.2	C27—C272—H27D	109.5
C12—C13—H13	119.2	C27—C272—H27E	109.5
C15—C14—C13	119.8 (2)	H27D—C272—H27E	109.5
C15—C14—H14	120.1	C27—C272—H27F	109.5
C13—C14—H14	120.1	H27D—C272—H27F	109.5
C14—C15—C16	121.4 (2)	H27E—C272—H27F	109.5
C14—C15—H15	119.3	C28—C281—H28A	109.5
C16—C15—H15	119.3	C28—C281—H28B	109.5
C15—C16—C11	117.5 (2)	H28A—C281—H28B	109.5
C15—C16—C18	120.7 (2)	C28—C281—H28C	109.5
C11—C16—C18	121.8 (2)	H28A—C281—H28C	109.5
C171—C17—C12	111.0 (2)	H28B—C281—H28C	109.5
C171—C17—C172	109.0 (2)	C38—C381—H38A	109.5
C12—C17—C172	113.7 (2)	C38—C381—H38B	109.5
C171—C17—H17	107.6	H38A—C381—H38B	109.5
C12—C17—H17	107.6	C38—C381—H38C	109.5
C172—C17—H17	107.6	H38A—C381—H38C	109.5
C181—C18—C182	109.8 (3)	H38B—C381—H38C	109.5
C181—C18—C16	111.7 (2)	C38—C382—H38D	109.5
C182—C18—C16	111.0 (3)	C38—C382—H38E	109.5
C181—C18—H18	108.1	H38D—C382—H38E	109.5
C182—C18—H18	108.1	C38—C382—H38F	109.5
C16—C18—H18	108.1	H38D—C382—H38F	109.5
C22—C21—C26	122.77 (19)	H38E—C382—H38F	109.5
C22—C21—N2	118.44 (19)	C47—C471—H47A	109.5
C26—C21—N2	118.69 (18)	C47—C471—H47B	109.5
C23—C22—C21	117.2 (2)	H47A—C471—H47B	109.5
C23—C22—C27	120.9 (2)	C47—C471—H47C	109.5
C21—C22—C27	121.88 (19)	H47A—C471—H47C	109.5
C24—C23—C22	121.5 (2)	H47B—C471—H47C	109.5
C24—C23—H23	119.3	C47—C472—H47D	109.5
C22—C23—H23	119.3	C47—C472—H47E	109.5
C25—C24—C23	119.9 (2)	H47D—C472—H47E	109.5
C25—C24—H24	120.0	C47—C472—H47F	109.5
C23—C24—H24	120.0	H47D—C472—H47F	109.5
C24—C25—C26	121.3 (2)	H47E—C472—H47F	109.5
C24—C25—H25	119.4	C28—C282—H28D	109.5
C26—C25—H25	119.4	C28—C282—H28E	109.5
C25—C26—C21	117.3 (2)	H28D—C282—H28E	109.5
C25—C26—C28	120.9 (2)	C28—C282—H28F	109.5
C21—C26—C28	121.81 (19)	H28D—C282—H28F	109.5
C22—C27—C272	110.4 (2)	H28E—C282—H28F	109.5
C22—C27—C271	112.7 (2)	C37—C372—H37A	109.5
C272—C27—C271	110.2 (2)	C37—C372—H37B	109.5
C22—C27—H27	107.8	H37A—C372—H37B	109.5
C272—C27—H27	107.8	C37—C372—H37C	109.5
C271—C27—H27	107.8	H37A—C372—H37C	109.5
C26—C28—C281	110.2 (2)	H37B—C372—H37C	109.5
C26—C28—C282	112.6 (2)	C37—C371—H37D	109.5
C281—C28—C282	110.2 (2)	C37—C371—H37E	109.5
C26—C28—H28	107.9	H37D—C371—H37E	109.5
C281—C28—H28	107.9	C37—C371—H37F	109.5
C282—C28—H28	107.9	H37D—C371—H37F	109.5
C4—N3—C5	111.83 (18)	H37E—C371—H37F	109.5
C4—N3—C31	125.68 (18)	C18—C182—H18D	109.5
C5—N3—C31	122.35 (18)	C18—C182—H18E	109.5
C4—N4—C6	111.76 (17)	H18D—C182—H18E	109.5
C4—N4—C41	126.80 (17)	C18—C182—H18F	109.5
C6—N4—C41	121.44 (18)	H18D—C182—H18F	109.5
N4—C4—N3	103.43 (17)	H18E—C182—H18F	109.5
N4—C4—Ag1	127.26 (14)	C48—C481—H48A	109.5
N3—C4—Ag1	129.28 (14)	C48—C481—H48B	109.5
C6—C5—N3	106.4 (2)	H48A—C481—H48B	109.5
C6—C5—H5	126.8	C48—C481—H48C	109.5
N3—C5—H5	126.8	H48A—C481—H48C	109.5
C5—C6—N4	106.5 (2)	H48B—C481—H48C	109.5
C5—C6—H6	126.7	C48—C482—H48D	109.5
N4—C6—H6	126.7	C48—C482—H48E	109.5
C36—C31—C32	123.8 (2)	H48D—C482—H48E	109.5
C36—C31—N3	118.6 (2)	C48—C482—H48F	109.5
C32—C31—N3	117.5 (2)	H48D—C482—H48F	109.5
C33—C32—C31	116.9 (2)	H48E—C482—H48F	109.5
C33—C32—C37	121.0 (2)	C48—C483—H48G	109.5
C31—C32—C37	122.1 (2)	C48—C483—H48H	109.5
C34—C33—C32	120.7 (2)	H48G—C483—H48H	109.5
C34—C33—H33	119.6	C48—C483—H48I	109.5
C32—C33—H33	119.6	H48G—C483—H48I	109.5
C33—C34—C35	120.9 (2)	H48H—C483—H48I	109.5
C33—C34—H34	119.6	C48—C484—H48J	109.5
C35—C34—H34	119.6	C48—C484—H48K	109.5
C34—C35—C36	120.5 (2)	H48J—C484—H48K	109.5
C34—C35—H35	119.7	C48—C484—H48L	109.5
C36—C35—H35	119.7	H48J—C484—H48L	109.5
C31—C36—C35	117.1 (2)	H48K—C484—H48L	109.5
C31—C36—C38	122.5 (2)	C72—O71—C75	86.1 (6)
C35—C36—C38	120.4 (2)	C73—C72—O71	107.3 (6)
C372—C37—C32	111.8 (3)	C73—C72—H72A	110.2
C372—C37—C371	111.7 (3)	O71—C72—H72A	110.2
C32—C37—C371	110.5 (3)	C73—C72—H72B	110.2
C372—C37—H37	107.6	O71—C72—H72B	110.2
C32—C37—H37	107.6	H72A—C72—H72B	108.5
C371—C37—H37	107.5	C72—C73—C74	105.8 (6)
C36—C38—C382	111.2 (2)	C72—C73—H73A	110.6
C36—C38—C381	110.9 (2)	C74—C73—H73A	110.6
C382—C38—C381	110.1 (2)	C72—C73—H73B	110.6
C36—C38—H38	108.2	C74—C73—H73B	110.6
C382—C38—H38	108.2	H73A—C73—H73B	108.7
C381—C38—H38	108.2	C75—C74—C73	82.9 (7)
C46—C41—C42	123.9 (2)	C75—C74—H74A	114.8
C46—C41—N4	117.73 (19)	C73—C74—H74A	114.8
C42—C41—N4	118.2 (2)	C75—C74—H74B	114.8
C41—C42—C43	117.1 (2)	C73—C74—H74B	114.8
C41—C42—C47	122.2 (2)	H74A—C74—H74B	111.9
C43—C42—C47	120.7 (2)	C74—C75—O71	101.8 (8)
C44—C43—C42	120.4 (2)	C74—C75—H75A	111.4
C44—C43—H43	119.8	O71—C75—H75A	111.4
C42—C43—H43	119.8	C74—C75—H75B	111.4
C43—C44—C45	121.1 (2)	O71—C75—H75B	111.4
C43—C44—H44	119.5	H75A—C75—H75B	109.3
C45—C44—H44	119.5	C72'—O71'—C75'	84.2 (6)
C44—C45—C46	120.9 (2)	O71'—C72'—C73'	109.5 (8)
C44—C45—H45	119.6	O71'—C72'—H72C	109.8
C46—C45—H45	119.6	C73'—C72'—H72C	109.8
C41—C46—C45	116.7 (2)	O71'—C72'—H72D	109.8
C41—C46—C48	122.3 (2)	C73'—C72'—H72D	109.8
C45—C46—C48	121.1 (2)	H72C—C72'—H72D	108.2
C471—C47—C42	111.4 (3)	C72'—C73'—C74'	99.8 (6)
C471—C47—C472	109.0 (3)	C72'—C73'—H73C	111.8
C42—C47—C472	112.0 (2)	C74'—C73'—H73C	111.8
C471—C47—H47	108.1	C72'—C73'—H73D	111.8
C42—C47—H47	108.1	C74'—C73'—H73D	111.8
C472—C47—H47	108.1	H73C—C73'—H73D	109.5
C482—C48—C46	110.4 (4)	C75'—C74'—C73'	77.9 (5)
C483—C48—C46	111.5 (4)	C75'—C74'—H74C	115.6
C482—C48—C481	116.3 (7)	C73'—C74'—H74C	115.6
C46—C48—C481	112.3 (4)	C75'—C74'—H74D	115.6
C483—C48—C484	109.8 (5)	C73'—C74'—H74D	115.6
C46—C48—C484	112.5 (4)	H74C—C74'—H74D	112.6
C482—C48—H48	105.7	C74'—C75'—O71'	97.0 (7)
C46—C48—H48	105.7	C74'—C75'—H75C	112.4
C481—C48—H48	105.7	O71'—C75'—H75C	112.4
C483—C48—H48'	107.6	C74'—C75'—H75D	112.4
C46—C48—H48'	107.6	O71'—C75'—H75D	112.4
C484—C48—H48'	107.6	H75C—C75'—H75D	109.9
C17—C171—H17A	109.5		
			
C2—N1—C1—N2	1.0 (2)	C31—N3—C4—Ag1	6.6 (3)
C11—N1—C1—N2	178.67 (19)	C4—N3—C5—C6	−0.3 (3)
C2—N1—C1—Ag1	−178.36 (15)	C31—N3—C5—C6	175.7 (2)
C11—N1—C1—Ag1	−0.6 (3)	N3—C5—C6—N4	−0.2 (3)
C3—N2—C1—N1	−0.8 (2)	C4—N4—C6—C5	0.7 (3)
C21—N2—C1—N1	177.90 (19)	C41—N4—C6—C5	−179.5 (2)
C3—N2—C1—Ag1	178.49 (16)	C4—N3—C31—C36	−101.7 (3)
C21—N2—C1—Ag1	−2.8 (3)	C5—N3—C31—C36	82.9 (3)
C1—N1—C2—C3	−0.8 (3)	C4—N3—C31—C32	81.9 (3)
C11—N1—C2—C3	−178.6 (2)	C5—N3—C31—C32	−93.5 (3)
N1—C2—C3—N2	0.2 (3)	C36—C31—C32—C33	2.1 (4)
C1—N2—C3—C2	0.4 (3)	N3—C31—C32—C33	178.2 (2)
C21—N2—C3—C2	−178.4 (2)	C36—C31—C32—C37	−175.8 (2)
C1—N1—C11—C16	−93.8 (3)	N3—C31—C32—C37	0.4 (3)
C2—N1—C11—C16	83.7 (3)	C31—C32—C33—C34	−1.3 (4)
C1—N1—C11—C12	91.0 (3)	C37—C32—C33—C34	176.6 (3)
C2—N1—C11—C12	−91.5 (2)	C32—C33—C34—C35	−0.3 (4)
C16—C11—C12—C13	0.8 (3)	C33—C34—C35—C36	1.3 (4)
N1—C11—C12—C13	175.8 (2)	C32—C31—C36—C35	−1.1 (3)
C16—C11—C12—C17	−179.8 (2)	N3—C31—C36—C35	−177.3 (2)
N1—C11—C12—C17	−4.8 (3)	C32—C31—C36—C38	178.6 (2)
C11—C12—C13—C14	−2.1 (4)	N3—C31—C36—C38	2.5 (3)
C17—C12—C13—C14	178.5 (2)	C34—C35—C36—C31	−0.6 (4)
C12—C13—C14—C15	1.6 (4)	C34—C35—C36—C38	179.6 (2)
C13—C14—C15—C16	0.4 (4)	C33—C32—C37—C372	60.4 (4)
C14—C15—C16—C11	−1.6 (4)	C31—C32—C37—C372	−121.9 (3)
C14—C15—C16—C18	179.1 (3)	C33—C32—C37—C371	−64.6 (4)
C12—C11—C16—C15	1.0 (3)	C31—C32—C37—C371	113.1 (3)
N1—C11—C16—C15	−174.0 (2)	C31—C36—C38—C382	129.5 (2)
C12—C11—C16—C18	−179.7 (2)	C35—C36—C38—C382	−50.7 (3)
N1—C11—C16—C18	5.3 (3)	C31—C36—C38—C381	−107.6 (3)
C13—C12—C17—C171	89.4 (3)	C35—C36—C38—C381	72.1 (3)
C11—C12—C17—C171	−90.0 (3)	C4—N4—C41—C46	98.9 (3)
C13—C12—C17—C172	−34.1 (3)	C6—N4—C41—C46	−80.9 (3)
C11—C12—C17—C172	146.6 (3)	C4—N4—C41—C42	−85.9 (3)
C15—C16—C18—C181	63.9 (4)	C6—N4—C41—C42	94.3 (3)
C11—C16—C18—C181	−115.4 (3)	C46—C41—C42—C43	0.8 (3)
C15—C16—C18—C182	−59.0 (4)	N4—C41—C42—C43	−174.10 (19)
C11—C16—C18—C182	121.7 (4)	C46—C41—C42—C47	179.8 (2)
C1—N2—C21—C22	94.2 (2)	N4—C41—C42—C47	4.9 (3)
C3—N2—C21—C22	−87.2 (3)	C41—C42—C43—C44	0.3 (3)
C1—N2—C21—C26	−89.2 (3)	C47—C42—C43—C44	−178.7 (2)
C3—N2—C21—C26	89.4 (2)	C42—C43—C44—C45	−1.3 (4)
C26—C21—C22—C23	2.9 (3)	C43—C44—C45—C46	1.3 (4)
N2—C21—C22—C23	179.34 (19)	C42—C41—C46—C45	−0.8 (3)
C26—C21—C22—C27	−178.2 (2)	N4—C41—C46—C45	174.13 (19)
N2—C21—C22—C27	−1.7 (3)	C42—C41—C46—C48	178.9 (2)
C21—C22—C23—C24	−1.2 (3)	N4—C41—C46—C48	−6.2 (3)
C27—C22—C23—C24	179.9 (2)	C44—C45—C46—C41	−0.3 (3)
C22—C23—C24—C25	−0.8 (4)	C44—C45—C46—C48	−179.9 (2)
C23—C24—C25—C26	1.2 (4)	C41—C42—C47—C471	−89.0 (3)
C24—C25—C26—C21	0.4 (3)	C43—C42—C47—C471	89.9 (3)
C24—C25—C26—C28	179.4 (2)	C41—C42—C47—C472	148.6 (2)
C22—C21—C26—C25	−2.5 (3)	C43—C42—C47—C472	−32.4 (3)
N2—C21—C26—C25	−178.97 (18)	C41—C46—C48—C482	108.1 (9)
C22—C21—C26—C28	178.5 (2)	C45—C46—C48—C482	−72.3 (9)
N2—C21—C26—C28	2.1 (3)	C41—C46—C48—C483	−86.4 (6)
C23—C22—C27—C272	81.1 (3)	C45—C46—C48—C483	93.2 (6)
C21—C22—C27—C272	−97.8 (3)	C41—C46—C48—C481	−120.4 (9)
C23—C22—C27—C271	−42.6 (3)	C45—C46—C48—C481	59.2 (9)
C21—C22—C27—C271	138.5 (2)	C41—C46—C48—C484	149.7 (6)
C25—C26—C28—C281	−75.7 (3)	C45—C46—C48—C484	−30.6 (6)
C21—C26—C28—C281	103.2 (2)	C75—O71—C72—C73	−45.6 (8)
C25—C26—C28—C282	47.8 (3)	O71—C72—C73—C74	12.0 (9)
C21—C26—C28—C282	−133.3 (2)	C72—C73—C74—C75	31.5 (9)
C6—N4—C4—N3	−0.8 (3)	C73—C74—C75—O71	−63.1 (8)
C41—N4—C4—N3	179.4 (2)	C72—O71—C75—C74	75.7 (9)
C6—N4—C4—Ag1	177.48 (18)	C75'—O71'—C72'—C73'	46.5 (9)
C41—N4—C4—Ag1	−2.3 (3)	O71'—C72'—C73'—C74'	−8.2 (11)
C5—N3—C4—N4	0.7 (3)	C73'—C74'—C75'—O71'	70.7 (6)
C31—N3—C4—N4	−175.1 (2)	C72'—O71'—C75'—C74'	−84.5 (7)
C5—N3—C4—Ag1	−177.59 (18)		

### Hydrogen-bond geometry (Å, º)

Cg1, Cg2 and Cg3 are the centroids of rings C31–C26, C11–C16 and C21–C26, respectively.

**Table:**

*D*—H···*A*	*D*—H	H···*A*	*D*···*A*	*D*—H···*A*
C5—H5···O71	0.95	2.42	3.292 (5)	153
C3—H3···Cl1	0.95	2.51	3.422 (2)	161
C35—H35···Cl1^i^	0.95	2.68	3.627 (3)	174
C43—H43···Cl1^ii^	0.95	2.64	3.562 (2)	163
C171—H17*B*···*Cg*1	0.98	2.81	3.532 (4)	131
C372—H37*C*···*Cg*2	0.98	2.94	3.613 (4)	126
C481—H48*A*···*Cg*3	0.98	2.98	3.840 (12)	147

Symmetry codes: (i) *x*+1/2, −*y*+3/2, *z*+1/2; (ii) *x*+1, *y*, *z*.
